# Supplementary material for: Life expectancy changes since COVID-19
Source: Nat Hum Behav. 2022 Oct 17;6(12):1649–59. doi: 10.1038/s41562-022-01450-3 (PMC9755047; doi:10.1038/s41562-022-01450-3)
Supplement: Supplementary file 1 — Supplementary Tables 1–4 and Fig. 1. [file 41562_2022_1450_MOESM1_ESM.pdf]

nature human behaviour

Article

https://doi.org/10.1038/s41562-022-01450-3

Life expectancy changes since COVID-19

In the format provided by the authors and unedited

Supplementary Tables

Supplementary Table 1: Months of female life expectancy (LE) changes and deficits (labelled ES) since the start of the pandemic attributed to age-specific mortality changes (labelled AT). LE deficit is defined as observed minus expected life expectancy had pre-pandemic mortality trends continued.

	Net LE diff 2019 to 21			LE changes 2020			LE changes 2021			LE deficit 2021		
	AT[1]	ES[2]	CI[3]	AT	ES	CI	AT	ES	CI	AT	ES	CI
AUT	$\downarrow^{\mathbf{60+}}$	−5.0	[−6.7; −3.4]	$\downarrow^{60+}$	−6.7	[−8.3; −4.9]	$\uparrow^{60+}$	1.7	[0.0; 3.4]	$\downarrow^{60+}$	−8.4	[−6.8; −10.2]
BEL	$\uparrow^{<60}$	1.2	[−0.2; 2.6]	$\downarrow^{60+}$	−11.1	[−12.8; −9.5]	$\uparrow^{60+}$	12.3	[10.6; 13.8]	$\downarrow^{60+}$	−3.9	[−2.2; −5.5]
BGR	$\downarrow^{60+}$	−42.3	[−44.5; −40.1]	$\downarrow^{60+}$	−15.0	[−17.3; −12.9]	$\downarrow^{60+}$	−27.3	[−29.2; −25.0]	$\downarrow^{60+}$	−43.1	[−41.0; −45.4]
CHE	$\uparrow^{60+}$	1.3	[−0.1; 3.0]	$\downarrow^{60+}$	−5.6	[−7.0; −3.8]	$\uparrow^{60+}$	6.9	[5.4; 8.6]	$\downarrow^{60+}$	−2.8	[−1.0; −4.8]
CHL	$\downarrow^{60+}$	−17.6	[−19.3; −16.0]	$\downarrow^{60+}$	−9.4	[−11.0; −8.0]	$\downarrow^{60+}$	−8.2	[−9.7; −6.8]	$\downarrow^{60+}$	−20.7	[−18.7; −22.2]
CZE	$\downarrow^{60+}$	−17.5	[−19.5; −15.7]	$\downarrow^{60+}$	−9.4	[−11.1; −7.9]	$\downarrow^{60+}$	−8.1	[−9.8; −6.6]	$\downarrow^{60+}$	−21.2	[−19.8; −22.9]
DEU	$\downarrow^{60+}$	−3.9	[−4.5; −3.4]	$\downarrow^{\mathbf{60+}}$	−1.7	[−2.3; −1.2]	$\downarrow^{60+}$	−2.2	[−2.8; −1.6]	$\downarrow^{60+}$	−8.0	[−7.5; −8.7]
DNK	$\downarrow^{60+}$	−1.6	[−3.5; 0.6]	$\uparrow^{60+}$	1.1	[−0.6; 3.9]	$\downarrow^{60+}$	−2.7	[−4.5; −0.3]	$\downarrow^{60+}$	−3.9	[−1.3; −6.2]
EST	$\downarrow^{60+}$	−18.6	[−22.5; −14.4]	$\downarrow^{60+}$	−1.5	[−6.6; 2.6]	$\downarrow^{60+}$	−17.1	[−21.6; −12.4]	$\downarrow^{60+}$	−23.3	[−18.1; −28.9]
ESP	$\downarrow^{60+}$	−5.5	[−6.2; −4.7]	$\downarrow^{60+}$	−14.1	[−15.0; −13.4]	$\uparrow^{\mathbf{60+}}$	8.7	[7.8; 9.4]	$\downarrow^{60+}$	−11.1	[−10.4; −12.0]
FIN	$\downarrow^{\mathbf{60+}}$	−0.6	[−2.8; 2.1]	$\uparrow^{\mathbf{<60}}$	0.9	[−1.3; 3.3]	$\downarrow^{<60}$	−1.6	[−3.5; 0.7]	$\downarrow^{\mathbf{60+}}$	−2.8	[−0.5; −4.9]
FRA	$\uparrow^{\mathbf{<60}}$	0.1	[−0.9; 0.9]	$\downarrow^{\mathbf{60+}}$	−5.0	[−5.8; −4.3]	$\uparrow^{60+}$	5.1	[4.4; 5.9]	$\downarrow^{\mathbf{60+}}$	−2.2	[−1.3; −2.9]
EAW	$\downarrow^{60+}$	−7.0	[−7.7; −6.3]	$\downarrow^{60+}$	−9.4	[−10.2; −8.7]	$\uparrow^{\mathbf{60+}}$	2.4	[1.5; 3.2]	$\downarrow^{60+}$	−10.6	[−9.7; −11.5]
NIR	$\downarrow^{60+}$	−8.3	[−13.2; −3.7]	$\downarrow^{60+}$	−8.4	[−12.1; −5.2]	$\uparrow^{\mathbf{60+}}$	0.0	[−3.7; 4.4]	$\downarrow^{60+}$	−11.4	[−7.2; −15.9]
SCT	$\downarrow^{60+}$	−8.4	[−11.2; −6.3]	$\downarrow^{60+}$	−5.4	[−8.0; −3.2]	$\downarrow^{\mathbf{<60}}$	−3.0	[−4.8; −0.4]	$\downarrow^{60+}$	−8.3	[−5.7; −10.6]
GRC	$\downarrow^{60+}$	−12.4	[−14.2; −11.2]	$\downarrow^{\mathbf{60+}}$	−2.5	[−4.1; −0.5]	$\downarrow^{60+}$	−10.0	[−11.7; −8.3]	$\downarrow^{\mathbf{60+}}$	−11.0	[−9.2; −12.6]
HRV	$\downarrow^{60+}$	−19.7	[−21.8; −17.4]	$\downarrow^{60+}$	−8.8	[−11.2; −6.6]	$\downarrow^{60+}$	−10.9	[−13.2; −8.3]	$\downarrow^{60+}$	−25.7	[−23.4; −28.3]
HUN	$\downarrow^{60+}$	−21.5	[−23.1; −19.8]	$\downarrow^{60+}$	−7.5	[−9.2; −5.8]	$\downarrow^{60+}$	−14.1	[−15.5; −12.4]	$\downarrow^{60+}$	−26.4	[−24.7; −28.0]
ISL	$\downarrow^{60+}$	−3.7	[−13.9; 4.6]	$\downarrow^{<60}$	−3.7	[−13.6; 5.2]	$\downarrow^{\mathbf{60+}}$	0.0	[−10.1; 8.4]	$\downarrow^{\mathbf{60+}}$	−4.7	[6.2; −15.8]
ITA	$\downarrow^{60+}$	−6.0	[−6.6; −5.4]	$\downarrow^{60+}$	−10.0	[−10.7; −9.4]	$\uparrow^{\mathbf{60+}}$	4.0	[3.4; 4.6]	$\downarrow^{60+}$	−11.6	[−10.9; −12.4]
LTU	$\downarrow^{60+}$	−26.0	[−29.7; −22.8]	$\downarrow^{60+}$	−14.2	[−18.0; −10.9]	$\downarrow^{60+}$	−11.8	[−15.3; −9.0]	$\downarrow^{60+}$	−35.7	[−32.7; −38.8]
NLD	$\downarrow^{60+}$	−6.2	[−7.4; −4.8]	$\downarrow^{60+}$	−5.7	[−6.9; −4.2]	$\downarrow^{\mathbf{<60}}$	−0.5	[−1.8; 0.8]	$\downarrow^{60+}$	−8.8	[−7.5; −10.1]
NOR	$\uparrow^{\mathbf{<60}}$	0.3	[−2.6; 2.5]	$\uparrow^{60+}$	2.1	[−0.1; 4.0]	$\downarrow^{\mathbf{60+}}$	−1.8	[−3.8; 0.7]	$\downarrow^{60+}$	−2.5	[0.0; −4.7]
POL	$\downarrow^{60+}$	−23.8	[−24.6; −22.9]	$\downarrow^{60+}$	−10.5	[−11.4; −9.6]	$\downarrow^{60+}$	−13.3	[−14.1; −12.6]	$\downarrow^{60+}$	−27.1	[−26.3; −27.9]
PRT	$\downarrow^{60+}$	−5.7	[−7.4; −3.8]	$\downarrow^{60+}$	−6.9	[−8.7; −5.2]	$\uparrow^{\mathbf{60+}}$	1.1	[−0.8; 2.6]	$\downarrow^{\mathbf{60+}}$	−7.8	[−6.3; −9.4]
SWE	$\uparrow^{60+}$	1.5	[−0.1; 3.2]	$\downarrow^{\mathbf{60+}}$	−5.3	[−6.6; −3.8]	$\uparrow^{60+}$	6.8	[5.2; 8.5]	$\downarrow^{\mathbf{60+}}$	−2.6	[−1.0; −4.2]
SVN	$\downarrow^{60+}$	−4.1	[−7.2; −0.7]	$\downarrow^{60+}$	−10.2	[−13.2; −6.4]	$\uparrow^{60+}$	6.1	[2.7; 9.2]	$\downarrow^{60+}$	−7.7	[−4.5; −11.0]
SVK	$\downarrow^{60+}$	−30.3	[−32.6; −28.1]	$\downarrow^{60+}$	−8.5	[−11.2; −6.5]	$\downarrow^{60+}$	−21.8	[−24.2; −19.5]	$\downarrow^{60+}$	−35.6	[−33.4; −38.4]
USA	$\downarrow^{<60}$	−21.4	[−22.2; −20.4]	$\downarrow^{60+}$	−22.6	[−23.3; −21.9]	$\uparrow^{\mathbf{60+}}$	1.2	[0.4; 2.0]	$\downarrow^{60+}$	−28.1	[−27.0; −29.0]

[1] Attribution of life expectancy changes to
mortality *increases* among primarily $\downarrow^{60+}$, solely $\downarrow^{\mathbf{60+}}$, primarily $\downarrow^{<60}$, solely $\downarrow^{\mathbf{<60}}$,
mortality *decreases* among primarily $\uparrow^{60+}$, solely $\uparrow^{\mathbf{60+}}$, primarily $\uparrow^{<60}$, solely $\uparrow^{\mathbf{<60}}$.
[2] Central estimate in months
[3] 95% confidence interval

Supplementary Table 2: Months of male life expectancy (LE) changes and deficits (labelled ES) since the start of the pandemic attributed to age-specific mortality changes (labelled AT). LE deficit is defined as observed minus expected life expectancy had pre-pandemic mortality trends continued.

	Net LE diff 2019 to 21			LE changes 2020			LE changes 2021			LE deficit 2021		
	AT[1]	ES[2]	CI[3]	AT	ES	CI	AT	ES	CI	AT	ES	CI
AUT	$\downarrow^{60+}$	−9.6	[−11.4; −7.4]	$\downarrow^{60+}$	−9.1	[−10.7; −7.0]	$\downarrow^{\mathbf{<60}}$	−0.5	[−2.4; 1.3]	$\downarrow^{60+}$	−15.1	[−13.2; −17.2]
BEL	$\downarrow^{60+}$	−3.1	[−4.6; −1.1]	$\downarrow^{60+}$	−12.2	[−14.1; −10.7]	$\uparrow^{60+}$	9.1	[7.8; 10.6]	$\downarrow^{60+}$	−9.4	[−7.9; −11.0]
BGR	$\downarrow^{60+}$	−41.2	[−43.8; −38.3]	$\downarrow^{60+}$	−19.0	[−21.4; −16.5]	$\downarrow^{60+}$	−22.2	[−24.2; −20.3]	$\downarrow^{60+}$	−43.4	[−41.5; −45.9]
CHE	$\downarrow^{<60}$	−1.6	[−3.2; 0.1]	$\downarrow^{60+}$	−10.0	[−12.0; −8.1]	$\uparrow^{60+}$	8.4	[6.6; 10.2]	$\downarrow^{60+}$	−8.9	[−6.7; −10.7]
CHL	$\downarrow^{60+}$	−22.7	[−24.3; −21.0]	$\downarrow^{60+}$	−15.6	[−16.9; −14.2]	$\downarrow^{<60}$	−7.2	[−8.5; −5.6]	$\downarrow^{60+}$	−26.6	[−25.0; −28.2]
CZE	$\downarrow^{60+}$	−24.2	[−25.6; −22.7]	$\downarrow^{60+}$	−12.6	[−14.0; −11.0]	$\downarrow^{60+}$	−11.7	[−13.4; −10.2]	$\downarrow^{60+}$	−28.9	[−27.4; −30.6]
DEU	$\downarrow^{60+}$	−7.2	[−7.8; −6.6]	$\downarrow^{\mathbf{60+}}$	−3.3	[−3.9; −2.6]	$\downarrow^{<60}$	−3.8	[−4.4; −3.2]	$\downarrow^{60+}$	−12.3	[−11.7; −13.0]
DNK	$\uparrow^{\mathbf{<60}}$	1.0	[−1.3; 3.8]	$\uparrow^{60+}$	1.2	[−0.7; 3.3]	$\downarrow^{\mathbf{60+}}$	−0.2	[−2.2; 2.2]	$\downarrow^{60+}$	−2.0	[0.4; −4.8]
EST	$\downarrow^{60+}$	−25.1	[−30.4; −20.0]	$\downarrow^{<60}$	−1.6	[−6.5; 3.5]	$\downarrow^{60+}$	−23.4	[−27.8; −18.8]	$\downarrow^{60+}$	−31.8	[−26.9; −36.6]
ESP	$\downarrow^{60+}$	−8.6	[−9.6; −7.7]	$\downarrow^{60+}$	−15.1	[−15.9; −14.3]	$\uparrow^{\mathbf{60+}}$	6.5	[5.4; 7.3]	$\downarrow^{60+}$	−14.6	[−13.9; −15.7]
FIN	$\uparrow^{\mathbf{<60}}$	0.0	[−2.7; 2.4]	$\downarrow^{<60}$	−1.6	[−4.2; 0.9]	$\uparrow^{\mathbf{<60}}$	1.6	[−0.7; 4.3]	$\downarrow^{\mathbf{60+}}$	−3.9	[−1.5; −6.9]
FRA	$\downarrow^{\mathbf{60+}}$	−2.0	[−2.9; −1.1]	$\downarrow^{\mathbf{60+}}$	−6.8	[−7.7; −6.1]	$\uparrow^{60+}$	4.8	[3.9; 5.6]	$\downarrow^{60+}$	−6.4	[−5.5; −7.2]
EAW	$\downarrow^{60+}$	−11.1	[−11.9; −10.4]	$\downarrow^{60+}$	−12.8	[−13.7; −11.9]	$\uparrow^{\mathbf{60+}}$	1.7	[0.9; 2.7]	$\downarrow^{60+}$	−14.5	[−13.5; −15.3]
NIR	$\downarrow^{<60}$	−9.8	[−13.7; −5.6]	$\downarrow^{\mathbf{60+}}$	−8.2	[−12.0; −3.7]	$\downarrow^{\mathbf{<60}}$	−1.6	[−6.5; 2.7]	$\downarrow^{60+}$	−11.7	[−7.1; −15.8]
SCT	$\downarrow^{60+}$	−10.6	[−12.8; −8.4]	$\downarrow^{60+}$	−11.9	[−14.0; −9.7]	$\uparrow^{\mathbf{60+}}$	1.4	[−1.0; 3.5]	$\downarrow^{60+}$	−12.0	[−9.8; −14.5]
GRC	$\downarrow^{60+}$	−18.1	[−19.8; −16.2]	$\downarrow^{\mathbf{60+}}$	−3.8	[−5.9; −1.9]	$\downarrow^{60+}$	−14.3	[−16.0; −12.5]	$\downarrow^{60+}$	−21.4	[−19.4; −23.2]
HRV	$\downarrow^{60+}$	−20.8	[−23.4; −17.7]	$\downarrow^{60+}$	−9.5	[−12.7; −7.1]	$\downarrow^{60+}$	−11.3	[−14.1; −8.3]	$\downarrow^{60+}$	−26.1	[−24.0; −28.3]
HUN	$\downarrow^{60+}$	−25.6	[−27.6; −23.7]	$\downarrow^{60+}$	−8.5	[−10.5; −6.3]	$\downarrow^{60+}$	−17.1	[−19.1; −15.5]	$\downarrow^{60+}$	−30.5	[−28.7; −32.5]
ISL	$\downarrow^{\mathbf{<60}}$	−1.2	[−11.0; 9.6]	$\downarrow^{\mathbf{<60}}$	−3.2	[−14.8; 8.1]	$\uparrow^{\mathbf{60+}}$	2.0	[−7.2; 13.4]	$\downarrow^{\mathbf{<60}}$	−1.3	[8.9; −12.4]
ITA	$\downarrow^{60+}$	−8.3	[−9.0; −7.6]	$\downarrow^{60+}$	−14.2	[−15.0; −13.4]	$\uparrow^{\mathbf{60+}}$	5.9	[5.2; 6.6]	$\downarrow^{60+}$	−15.0	[−14.4; −15.7]
LTU	$\downarrow^{60+}$	−23.2	[−27.2; −18.9]	$\downarrow^{60+}$	−20.4	[−24.6; −16.3]	$\downarrow^{60+}$	−2.8	[−6.9; 0.5]	$\downarrow^{60+}$	−35.8	[−31.5; −40.1]
NLD	$\downarrow^{60+}$	−7.8	[−8.9; −6.5]	$\downarrow^{60+}$	−9.0	[−10.4; −7.5]	$\uparrow^{\mathbf{60+}}$	1.2	[−0.1; 2.2]	$\downarrow^{60+}$	−11.2	[−10.0; −12.5]
NOR	$\uparrow^{<60}$	3.2	[−0.1; 5.7]	$\uparrow^{60+}$	1.8	[−0.6; 4.5]	$\uparrow^{<60}$	1.3	[−1.2; 4.1]	$\downarrow^{\mathbf{60+}}$	−2.1	[1.0; −4.3]
POL	$\downarrow^{60+}$	−27.1	[−28.2; −26.0]	$\downarrow^{60+}$	−16.9	[−17.8; −15.8]	$\downarrow^{<60}$	−10.2	[−11.1; −9.2]	$\downarrow^{60+}$	−31.7	[−30.6; −32.8]
PRT	$\downarrow^{60+}$	−9.0	[−10.8; −7.2]	$\downarrow^{60+}$	−9.4	[−11.0; −7.6]	$\uparrow^{\mathbf{<60}}$	0.4	[−1.2; 2.3]	$\downarrow^{60+}$	−12.2	[−10.3; −13.8]
SWE	$\downarrow^{<60}$	−1.3	[−2.7; 0.3]	$\downarrow^{60+}$	−9.2	[−11.3; −7.5]	$\uparrow^{60+}$	7.9	[6.4; 9.6]	$\downarrow^{60+}$	−6.8	[−5.0; −8.3]
SVN	$\downarrow^{60+}$	−9.1	[−12.9; −4.5]	$\downarrow^{\mathbf{60+}}$	−9.4	[−14.1; −5.0]	$\uparrow^{\mathbf{60+}}$	0.3	[−3.7; 5.0]	$\downarrow^{60+}$	−10.5	[−6.1; −13.9]
SVK	$\downarrow^{60+}$	−33.5	[−36.0; −31.2]	$\downarrow^{60+}$	−9.5	[−11.7; −7.0]	$\downarrow^{60+}$	−23.9	[−26.3; −21.4]	$\downarrow^{60+}$	−40.5	[−38.1; −43.7]
USA	$\downarrow^{<60}$	−33.0	[−33.5; −32.6]	$\downarrow^{60+}$	−27.0	[−27.4; −26.5]	$\downarrow^{\mathbf{<60}}$	−6.0	[−6.5; −5.6]	$\downarrow^{<60}$	−36.0	[−35.5; −36.6]

[1]Attribution of life expectancy changes to
mortality *increases* among primarily $\downarrow^{60+}$, solely $\downarrow^{\mathbf{60+}}$, primarily $\downarrow^{<60}$, solely $\downarrow^{\mathbf{<60}}$,
mortality *decreases* among primarily $\uparrow^{60+}$, solely $\uparrow^{\mathbf{60+}}$, primarily $\uparrow^{<60}$, solely $\uparrow^{\mathbf{<60}}$.

[2]Central estimate in months

[3]95% confidence interval

Supplementary Table 3: Life expectancy losses and bounce-backs during six selected mortality shock events in the 20th century.

	**World War I (1914–1918)**				**Spanish Flu**			**World War II (1939–1945)**			
	PLE	TLL	TLC	YER	PLE	TLC	YER	PLE	TLL	TLC	YER
Austria	.	.	.	.	.	.	.	.	.	.	.
Belgium	.	.	.	.	.	.	.	60.1	−7.5	−1.8	1946
Bulgaria	.	.	.	.	.	.	.	.	.	.	.
Czech Republic	.	.	.	.	.	.	.	.	.	.	.
Denmark	58.9	−3.0	−2.7	1921	57.3	−1.0	1920	65.0	−1.7	1.1	no loss
Eng & Wal	53.8	−12.9	−12.9	1919	46.0	−5.1	1919	63.7	−5	−19	1946
Estonia	.	.	.	.	.	.	.	.	.	.	.
Finland	49.0	−16.9	−16.2	1921	46.5	−13.7	1920	57.2	−19.0	−0.4	1946
France	51.4	−22.9	−16.5	1920	43.0	−8.1	1919	58.9	−20.2	−4.0	1946
Hungary	.	.	.	.	.	.	.	.	.	.	.
Iceland	58.9	−15.0	−7.9	1926	59.0	−7.9	1926	65.0	−2.9	2.5	no loss
Italy	48.5	−24.0	−22.7	1921	38.1	−12.3	1919	56.2	−8.2	−1.3	1946
Lithuania	.	.	.	.	.	.	.	.	.	.	.
Latvia	.	.	.	.	.	.	.	.	.	.	.
Netherlands	57.4	−9.8	−9.7	1920	55.7	−8.0	1920	67.4	−12.6	−11.8	1946
N. Ireland	.	.	.	.	.	.	.	59.1	−2.9	4.3	no loss
Norway	58.3	−8.8	−8.0	1920	57.7	−7.4	1920	67.1	−1.9	1.1	no loss
Poland	.	.	.	.	.	.	.	.	.	.	.
Portugal	.	.	.	.	.	.	.	.	.	.	.
Russia	.	.	.	.	.	.	.	.	.	.	.
Scotland	51.5	−6.4	−2.6	1920	52.6	−3.8	1920	60.7	−3.6	2.3	no loss
Slovakia	.	.	.	.	.	.	.	.	.	.	.
Spain	42.6	−13.5	−12.2	1922	42.6	−12.2	1922	47.6	−1.8	10.2	no loss
Sweden	58.6	−10.6	−8.9	1920	58.8	−9.1	1920	65.5	−1.2	2.8	no loss
Switzerland	54.2	−10.2	−7.9	1918	55.8	−9.5	1921	63.8	−1.4	1.5	no loss
Ukraine	.	.	.	.	.	.	.	.	.	.	.
USA	.	.	.	.	.	.	.	62.4	−0.2	3.2	no loss

	**Influenza (1962)**				**Influenza (2015)**			**Soviet mortality crisis (1987–1995)**			
	PLE	TLL	TLC	YER	PLE	TLC	YER	PLE	TLL	TLC	YER
Austria	69.7	.	−0.2	1964	81.4	−0.2	2016			—	
Belgium	70.5	.	−0.3	1964	81.1	−0.2	2016			—	
Bulgaria	70.2	.	−0.7	1963	74.5	0.2	no loss			—	
Czech Republic	70.6	.	−0.7	1977	78.8	−0.2	2016			—	
Denmark	72.5	.	−0.1	1964	80.6	0.1	no loss			—	
Eng & Wal	71.0	.	0.0	no loss	81.4	−0.2	2017			—	
Estonia	69.6	.	0.2	no loss	77.1	0.6	no loss	70.9	−4.2	−3.1	2000
Finland	69.0	.	−0.3	1963	81.0	0.4	no loss			—	
France	71.0	.	−0.5	1964	82.5	−0.3	2017			—	
Hungary	69.0	.	−1.1	1964	75.9	−0.2	2016			—	
Iceland	73.4	.	0.2	no loss	82.7	−0.3	2018			—	
Italy	69.8	.	−0.6	1964	82.9	−0.4	2016			—	
Lithuania	70.5	.	−1.0	1963	74.6	−0.1	2016	72.4	−3.9	−3.4	2009
Latvia	.	.	.	.	.	.	.	71.0	−5.8	−5.0	2008
Netherlands	73.6	.	−0.3	1964	81.6	−0.2	2017			—	
N. Ireland	69.8	.	0.7	no loss	80.6	−0.3	2018			—	
Norway	73.6	.	−0.1	1964	82.1	0.2	no loss			—	
Poland	67.9	.	−0.3	1963	77.6	−0.2	2016			—	
Portugal	62.8	.	1.5	no loss	81.2	0.0	no loss			—	
Russia	.	.	.	.	.	.	.	69.9	−6.1	−5.3	2012
Scotland	69.1	.	0.1	no loss	79.4	−0.3	Ongoing			—	
Slovakia	70.8	.	−0.5	1964	76.9	−0.2	2016			—	
Spain	69.6	.	0.0	no loss	82.9	−0.2	2016			—	
Sweden	73.5	.	−0.1	1963	82.2	0.0	no loss			—	
Switzerland	71.7	.	−0.4	1964	.	.	.			—	
Ukraine	.	.	.	.	.	.	.	71.3	−4.5	−4.5	2013
USA	70.2	.	−0.1	1965	79.1	−0.1	2019			—	

Data by Human Mortality Database. (PLE) LE prior to the event; (TLL) Total LE loss over duration of event; (TLC) Total LE change over duration of event; (YER) Year of return to prior LE

Supplementary Table 4: Deviation of overall midyear population estimates (in 10,000) between UN World Population Prospect (WPP) and National Statistical Office (NSO) estimates.

	**2019 Population**			**2020 Population**			**2021 Population**		
	WPP	NSO	Dif[1]	WPP	NSO	Dif[1]	WPP	NSO	Dif[1]
AUT	895.5	887.9	-7.6	900.6	891.7	-9.0	904.3	896.1	-8.2
BEL	1153.9	1146.2	-7.7	1159.0	1150.7	-8.3			
BGR	700.0	697.6	-2.4	694.8	693.4	-1.4			
CHE	859.1	857.5	-1.6	865.5	863.8	-1.6	871.5	871.6	0.0
CHL	1895.2	1910.7	15.5	1911.6	1945.8	34.2	1921.2	1967.8	46.6
CZE	1068.9	1067.2	-1.7	1070.9	1069.8	-1.1			
DEU	8351.7	8309.3	-42.4	8378.4	8316.1	-62.3	8390.0	8331.9	-58.2
DNK	577.2	581.4	4.3	579.2	582.5	3.3	581.3	585.0	3.7
EAW	5924.6	5944.0	19.4	5937.0	5972.0	35.0	5947.9	5998.0	50.1
ESP	4673.7	4710.5	36.9	4675.5	4735.6	60.1	4674.5	4732.7	58.1
EST	132.6	132.7	0.1	132.7	132.9	0.3	132.5	132.6	0.1
FIN	553.2	552.2	-1.1	554.1	553.0	-1.1	554.8	554.0	-0.8
FRA	6513.0	6721.6	208.6	6527.4	6734.7	207.4			
GRC	1047.3	1072.2	24.8	1042.3	1161.8	119.5			
HRV	413.0	406.5	-6.5	410.5	404.8	-5.8			
HUN	968.5	977.1	8.6	966.0	975.0	9.0			
ISL	33.9	36.1	2.2	34.1	36.6	2.5	34.3	37.3	3.0
ITA	6055.0	5972.9	-82.1	6046.2	5943.9	-102.3	6036.7	5916.1	-120.7
LTU	276.0	279.4	3.5	272.2	279.5	7.3	269.0	278.7	9.7
NIR	189.0	189.4	0.4	189.7	189.6	-0.1	190.3	190.2	-0.2
NLD	1709.7	1734.5	24.8	1713.5	1744.2	30.7			
NOR	537.9	534.8	-3.1	542.1	538.0	-4.2	546.6	540.5	-6.1
POL	3788.8	3838.6	49.9	3784.7	3835.4	50.8	3779.7	3816.2	36.5
PRT	1022.6	1028.6	6.0	1019.7	1029.7	10.0			
SCO	543.7	546.3	2.6	543.1	546.6	3.5	542.4	546.9	4.6
SVK	545.7	545.4	-0.3	546.0	545.9	-0.1			
SVN	207.9	208.9	1.1	207.9	210.0	2.1	207.9	210.7	2.8
SWE	1003.6	1027.9	24.2	1009.9	1035.3	25.4	1016.0	1040.4	24.4
USA	32906.5	32833.0	-73.5	33100.3	32948.4	-151.9	33291.5	33499.8	208.3

[1]Differences between the WPP and NSO midyear population estimates.

Supplementary Figures

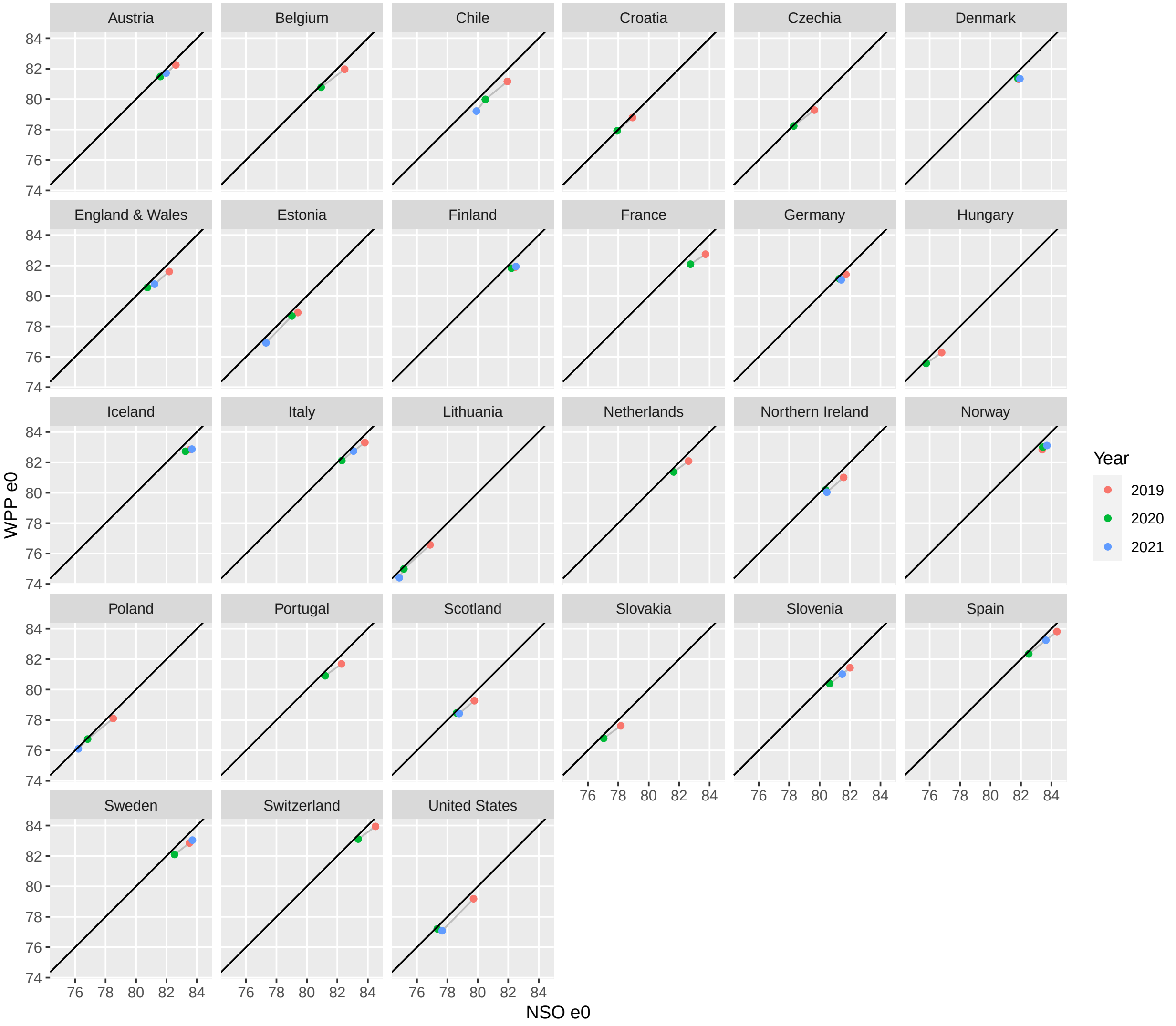

Supplementary Figure 1: Life expectancy (e0) estimates for 2019, 2020 and when available 2021, using population estimates from national statistical offices (NSOs) (x-axis) and UN World Population Prospects (WPP) (y-axis). Black line indicates x=y line.
